# Determinants of Community Integration Among Formerly Homeless Veterans Who Received Supportive Housing

**DOI:** 10.3389/fpsyt.2019.00472

**Published:** 2019-06-26

**Authors:** Melissa Chinchilla, Sonya Gabrielian, Gerhard Hellemann, Amy Glasmeier, Michael Green

**Affiliations:** ^1^VA HSR&D Center for the Study of Healthcare Innovation, Implementation, & Policy, Los Angeles, CA, United States; ^2^VA Greater Los Angeles Healthcare System, Los Angeles, CA, United States; ^3^Department of Psychiatry and Biobehavioral Sciences, University of California, Los Angeles, Los Angeles, CA, United States; ^4^Department of Urban Studies and Planning Massachusetts Institute of Technology, Cambridge, MA, United States

**Keywords:** homelessness, supportive housing, veterans, community integration, social integration, physical integration

## Abstract

Community integration is recognized as a meaningful goal that is highly relevant to the long-term success of supportive housing programs. Research to date highlights concerns that some individuals in permanent supportive housing remain socially isolated and have limited success in other domains of community integration. However, we know little about what factors impact formerly homeless veterans’ ability to achieve community integration. To identify factors associated with community integration among homeless veterans housed through the Department of Veterans Affairs’ (VA’s) Supportive Housing program (HUD-VASH), we performed secondary database analyses of Veterans (*n* = 560) housed *via* HUD-VASH in the VA Greater Los Angeles Healthcare System from 10/1/14–9/30/15. We conducted ordinal and logit regressions to examine associations between baseline HUD-VASH participant characteristics, supportive housing voucher type, health service utilization in the year post-housing, and three types of community integration outcomes (i.e., change in community adjustment, status of housing stability, and change in employment). Data were obtained from HOMES (VA’s homeless registry) and Corporate Data Warehouse (CDW) (VA’s a national administrative dataset of VA inpatient and outpatient care). Mental health service utilization was negatively associated with community adjustment, housing stability, and employment. Employment at baseline was positively associated with housing stability and employment. Also, substance use disorder visits was positively associated with employment, and combined medical and substance use disorder diagnoses was positively associated with change in community adjustment. We considered 29 variables and found relatively few were associated with community integration. Consistent with previous research, our study highlights the importance of mental health needs, and suggests that utilization of mental health services is an important indicator of improvements in community adjustment, housing stability, and employment.

On a single night in 2017, 553,742 people became homeless in the United States, and 40,056 of which were homeless veterans (1). The Department of Veterans Affairs (VA) has devoted tremendous resources toward addressing veteran homelessness (2). Between 2010 and 2017, homelessness among veterans declined by 46%, a decrease largely attributed to the crux of the VA’s homeless services: the U.S. Department of Housing and Urban Development (HUD)-VA Supportive Housing (VASH) program, which provides permanent, community-based housing and supportive services for homeless veterans (1). Housing, however, is only the first step toward addressing the community needs of this vulnerable population. After homeless veterans achieve housing, more fundamental needs grow important, e.g., involvement in the community.

The HUD-VASH is a permanent supportive housing (PSH) program (3, 4) that combines independent permanent housing with field-based case management and non-mandated linkages to health care (5, 6). HUD-VASH participants receive a “voucher” that subsidizes rental costs supplemented by 30% to 40% of the participant’s income (7). The HUD-VASH uses two types of vouchers: project-based and tenant-based. Project-based vouchers subsidize housing in dedicated multi-unit facilities for low-income persons. Tenant-based vouchers subsidize market rate housing in the community. The population served by the HUD-VASH is highly vulnerable, as the program targets chronically homeless persons who often have significant medical and mental health vulnerabilities and/or substance use disorders (SUD) (8). The VA’s progress in housing homeless veterans raises public health concerns about how to manage the long-lasting consequences of homelessness and poverty, e.g., housing maintenance, poor social networks, and limited vocational pursuits, once people are housed (9).

Research to date highlights concerns that some individuals in PSH remain socially isolated and have limited success in other domains of community integration (10, 11). Broadly, community integration refers to the way an individual is embedded in his or her community (12). One definition of community integration for PSH participants states that it consists of three dimensions: physical, social, and psychological. Physical integration involves time spent within one’s community and the ability to live independently. Social integration takes a social network perspective, examining social interactions, social roles, and social support. Psychological integration consists of a sense of belonging, including self-perception of community membership (13). Hence, community integration is typically defined as multi-dimensional, with components influencing one another. We focus on two dimensions of community integration that are available from the centralized database: social and physical.

Community integration has significant implications for mental and physical health (14). Social ties affect access to social and economic resources (15) and impact individuals’ health by impacting instrumental aid, information flows, and emotional support (16). Community integration is recognized as a meaningful goal that is highly relevant to the long-term success of PSH programs (10). Yet, little is known regarding what factors influence community integration for veterans placed in PSH.

Although substantial research examines factors that convey risk for experiencing homelessness, there are relatively fewer studies on individual characteristics and service utilization patterns that are associated with community integration among formerly homeless persons. Most research that have considered community integration among the formerly homeless specifically examined those with severe mental illness (17–19). This literature has shown that, among other factors, age (20, 21), race (21), physical and mental functioning (20), housing type (22–24), and service receipt (17, 20, 22) are associated with community integration.

Although the literature provides information on factors that affect community integration among homeless individuals with serious mental illness, very little is known regarding whether the same factors affect community integration among formerly homeless veterans. Homeless veterans have unique pathways that predispose them to homelessness, including combat exposure and military sexual trauma. Further, although veterans are able to access various VA benefits, some researchers speculate that post-deployment challenges outweigh the protective benefits offered through services and health-care access (25).

This paper focuses on community integration among formerly homeless veterans housed in the HUD-VASH. Community integration encompasses physical, social, and psychological integration (13). Given that the data available for these analyses are from centralized databases, we focused our analysis on two core themes: relationships with others (social integration) and independence in living (physical integration; 26). Using secondary data from the VA Greater Los Angeles (GLA), which boasts the nation’s largest HUD-VASH program, we identify individual characteristics, supportive housing voucher type (project-based vs. tenant-based), and health service utilization patterns associated with community integration outcomes (change in community adjustment, housing stability status, and change in employment).

## Methods

### Participants

We used the VA Homeless Operations Management and Evaluation System (HOMES), a centralized registry of the VA’s homeless service use, to identify participants enrolled in the HUD-VASH at the VA GLA (*n* = 1,117) over one fiscal year (from 10/1/14 to 9/30/15). We limited the sample to participants who achieved housing within 1 year (365 days) of program enrollment, noting that the nationwide HUD-VASH housing placement takes an average of 108 days from enrollment to housing placement (27). A total of 327 (29.3%) entries exceeded 1 year for placement and were removed from the dataset.

Using the HOMES quarterly status reports, we removed duplicate entries. We also resolved conflicting information; if a participant had two reports entered in one day and data entries did not match, the report was removed from our dataset. Duplicate entries and conflicting information led us to remove an additional 34 participants. Next, we assured that subjects had quarterly status report data for primary outcomes of interest, i.e., community adjustment, housing stability, and employment, which were derived using specified quarters. We removed 196 participants that did not contain data for any of our outcomes of interest.

For community adjustment, we included participants who had this variable for quarters 1 and 4 (*n* = 497). For housing stability, we included participants who had this information for quarter 4 (*n* = 506). Lastly, due to the low reporting for employment in general, particularly for quarter 4, we included participants who had ratings of employment for quarters 1 and 3 (*n* = 172). In total, our final sample consisted of 560 participants. Consequently, an additional 54 to 388 veterans were dropped depending on the analysis. The GLA Institutional Review Board approved all study activities.

### Independent Variables

We considered three types of variables that are associated with community integration in vulnerable populations: participant characteristics, e.g., demographics, (21, 22, 28), type of housing (24), and health service utilization (22, 28).

#### Participant Characteristics

The HOMES includes a baseline assessment (at the time of the VA’s homeless program entry) of participants’ demographic information as well as medical, mental health, and SUD diagnoses. We used the HOMES baseline assessment to obtain age, gender, race/ethnicity, years of education, employment status, presence or absence of children, relationship status (e.g., married or partnered versus single, divorced, or widowed), presence or absence of a criminal history, and number of homeless episodes. We used a baseline index of perceived physical health, which asked participants about their physical health at program entry, scored on a Likert scale from 1 (poor) to 5 (excellent). We also obtained data regarding the presence or absence of specific medical diagnoses, mental health diagnoses, and SUDs that are common among homeless persons (**Table 1**) (29–31). These diagnoses were collected from participants by case managers at the time of program entry. Health variables were combined to reflect co-occurring disorders (i.e., medical and mental health diagnoses, medical and SUD diagnoses, mental and SUD diagnoses, and all three types of diagnoses).

**Table 1:** Medical, mental, and addiction-related diagnoses.

Medical diagnoses	Mental health diagnoses	Addictive disorders	Diagnostic category
Chronic obstructive pulmonary disease	Bipolar disorder	Alcohol use disorder	At least one mental health diagnosis
Diabetes	Depressive disorder	Drug use disorder	At least one medical diagnosis
Heart disease	Adjustment disorder		At least one SUD
Stroke	Military post-traumatic stress disorder (PTSD)		No diagnosis
Chronic pain	Non-military PTSD		Combination of medical and mental health diagnoses
Seizures	Other anxiety disorders		Combination of medical and SUD diagnoses
Hepatitis C	Schizophrenia		Combination of mental and SUD diagnoses
HIV/AIDS	Other psychotic disorders		Combination of medical, mental, and SUD diagnoses
History of positive tuberculosis test	Personality disorders		
	Other psychiatric disorders		

#### Supportive Housing Voucher Type

Relevant to this study was voucher type (i.e., project-based versus tenant-based), which was used to determined the housing type. Voucher assignment was obtained from the HOMES and checked against the last known housing address, which was obtained from the VA’s Corporate Data Warehouse (CDW), a national administrative dataset of the VA’s inpatient and outpatient care. We used a list of addresses for the GLA HUD-VASH project-based sites to confirm the participants’ housing voucher type.

#### Service Utilization

We extracted health service utilization data from the CDW, including participants’ rates of the VA’s service utilization 1 year after housing placement. Date housed was used to determine the 1-year time frame after housing for each participant. We extracted the number of the HUD-VASH case management contacts, primary care visits, outpatient mental health visits, and outpatient SUD program visits. We also identified whether or not each participant had at least one emergency department visit, psychiatric hospitalization, and medical/surgical hospitalization.

### Outcomes

To assess community integration outcomes, we used the HOMES quarterly status reports completed by the HUD-VASH case managers (who performed supportive services) after housing placement. Case managers use quarterly reports to document a veteran’s progress in the HUD-VASH program. Per program requirements, a case manager must conduct at least one home visit per month. However, communication with case managers can occur more frequently based on the participant’s need. The completion of quarterly reports is intended to occur every 3 months; however, there is variability in time and rate of submission. We set a minimum of 30 days between reporting periods to assure that reports were standardized and reflected potential changes in community integration. Reports that were less than 30 days apart were removed from the database. We examined three outcome variables extracted from the quarterly reports: change in community adjustment (between quarters 1 and 4), status of housing stability (on quarter 4), and change in employment (between quarters 1 and 3). These outcomes were both captured in the HOMES and were part of our conceptual framework, which focused on social and physical integration by examining two core themes: relationships with others and independence in living. Community adjustment described participants’ relationships with their surrounding community, employment described both relationships and independence in living, and housing stability described independence in living (i.e., a participant’s ability to remain housed).

#### Community Adjustment

Community adjustment was captured on a five-point Likert scale ranging from 1 (greatly worsened) to 5 (greatly improved), which estimated community adjustment over the previous 90 days (i.e., how well a veteran is acclimating to his or her new neighborhood). This measure of community adjustment depicts a case manager’s assessment of how a veteran is engaging with the local community (e.g., use of resources and social relationships) and his or her ability to follow rental agreements. Case managers meet periodically with participants and are a primary contact for any challenges that arise with tenancy. Community adjustment reflects the core community integration domain of relationships with others (social integration). Data for quarter 4 were compared to data for quarter 1 to create an overall change score that assessed community adjustment over 1 year after housing (−1 = worsened, 0 = stayed the same, and 1 = improved).

#### Housing Stability

Housing stability was measured with a housing status variable stratified into one of three categories: obtained permanent housing without the HUD-VASH, retained housing with the HUD-VASH, or no longer in permanent housing. This variable was a proxy of the community integration domain of independent living (physical integration). We used entries from quarter 4 to create an ordinal outcome measure that assessed housing stability (0 = did not retain housing, 1 = retained HUD-VASH housing, and 2 = achieved permanent housing without the HUD-VASH). We only included participants who had a quarter 4 report; we could not verify if quarter 4 reports were omitted due to administrative oversight or loss of the HUD-VASH voucher.

#### Employment

Employment change compared employment status on quarters 1 and 3. These quarters were chosen for comparison due to the low reporting rates on quarter 4 (2.15%). We created a binary variable that captured if a participant’s employment trajectory was good (obtained a job or stayed employed) or poor (lost a job or remained unemployed) between quarters 1 and 3. Employment was included as part of our understanding of independent living (physical integration).

### Statistical Analysis

We used ordinal logistic regression models (for change in community adjustment and housing stability status) and a logistic regression model (for change in employment) to test the relationship between the independent variables and community integration outcomes. Ordinal logistic regression analyses were used to determine the odds ratio (OR) for outcome categories. Ordinal logistic regression uses the proportional odds assumption, meaning that the distances between pairs of categories (e.g., lowest versus next higher categories, then that category to the next one higher, and so on) are the same for each pair. To test this assumption, a likelihood ratio test was conducted and showed that the assumption was not violated.

For the employment outcome, we used logistic regression. Due to the low variability in employment outcomes and a small sample size due to low levels of reporting (see above), we were unable to statistically consider diagnoses as independent variables. Although not as precise as specific diagnoses, service utilization was used to assess medical vulnerability. In addition, we were not able to use mental health admission because only one participant reported such a hospitalization. For all independent variables, we reported a single summary score of effects (i.e., OR) with 95% confidence interval (95% CI). We ran three different logistic regressions. We used a Bonferroni correction that set the significance level to 0.017. It was not necessary to adjust for the various coefficients within each of the three regressions, as parameter estimates are conditioned on all parameters in the model. Therefore, *p* < 0.05 was chosen to assess the significance of coefficients in our models. All statistical analyses were performed using StataMP 14.

## Results

### Participant Characteristics

**Table 2** presents the baseline participant characteristics and service utilization over 1 year after housing. Most participants in our sample received tenant-based vouchers (88.39%), were middle-aged (mean age 52.93 years), male (93.57%), and single (87.98%), and self-identified as African American (54.17%) or White (38.18%). At baseline, participants reported high rates of unemployment (66.24%) and an average of 2.97 episodes of homelessness over the past 3 years. A notable proportion of participants reported at least one medical but no psychiatric diagnosis (15.18%) and a similar proportion reported at least one mental health and no medical diagnosis (15.89%). A total of 6.25% of participants reported at least one SUD without any comorbid medical or mental health diagnosis. Additionally, a significant number of participants reported co-occurring medical and mental health diagnoses (16.25%) or trimorbidity defined as co-occurring medical, mental health, and SUD diagnoses (11.79%). The rates of diagnoses are comparable to a prior study for which GLA HUD-VASH recipients received outpatient care (32).

**Table 2:** Participants’ characteristics and service utilization: VA GLA Healthcare System, 10/1/14–9/30/15.

	Mean (SD) or *n* (%), *n* = 560	Min	Max
Tenant-based voucher	495 (88.39%)		
Age	52.93 (12.95)	22.52	86.30
Male	524 (93.57%)		
Ethnicity			
Latino	91 (16.61%)		
Race			
African American	307 (57.17%)		
White	205 (38.18%)		
Married or partnered	66 (12.02%)		
Have children (yes)	143 (26.00%)		
Education (in years)	13.40 (1.82)	6	20
Employment			
Full-time	64 (11.79%)		
Military	11 (2.03%)		
Part-time	85 (15.65%)		
Student-vocational therapy	23 (4.24%)		
Unemployed	359 (66.24%)		
Criminal history (yes)	211 (39.44%)		
Homeless episodes (last 3 years)	2.97 (1.72)	0	5
Self-perceived physical health	2.59 (1.04)	1	5
Diagnostic category			
At least one mental health diagnosis	85 (15.18%)		
At least one medical diagnosis	89 (15.89%)		
At least one SUD^+^	35 (6.25%)		
No diagnosis	141 (25.18%)		
Combination of medical and mental health diagnoses	91 (16.25%)		
Combination of medical and SUD diagnoses	23 (4.11%)		
Combination of mental and SUD diagnoses	34 (6.07%)		
Combination of medical, mental, and SUD diagnoses	66 (11.79%)		
Service utilization^+^			
Primary care visits	8.22 (9.18)	0	72
Emergency department admission	171 (30.54%)		
Mental health visits	21.19 (34.06)	0	535
HUD-VASH visits	36.84 (24.47)	3	169
Outpatient SUD visits	1.08 (8.73)	0	140
Mental health inpatient admission	12 (2.14%)		
Medical/surgical inpatient admission	56 (10.00%)		

^+^Service utilization represents health behaviors 1 year after housing. SUD, substance use disorder; HUD-VASH, Department of Veterans Affairs’ (VA’s) Supportive Housing program.

Health service utilization in the year after housing placement is shown in **Table 2**. Nearly a third of participants had at least one emergency department visit (30.54%); fewer participants had at least one mental health admission (2.14%) or medical/surgical admission (10.00%). There was also notable utilization of the HUD-VASH case management (mean of 36.84 visits) as well as mental health visits (mean of 21.19) and primary care (mean of 8.22).

### Community Adjustment

**Table 3** shows the results of community adjustment ordinal logistic regression analyses. The community adjustment model as a whole was not statistically significant compared to a null model with no independent variables (likelihood ratio χ^2^ = 38.20, *p* = 0.12, pseudo-*R* ^2^ = 0.06). Nonetheless, the model identified two significant variables: combined medical and SUD diagnoses and mental health inpatient admissions. Combined medical and SUD diagnoses were associated with increased odds of improvement in community adjustment by 3.55, whereas a mental health inpatient admission was associated with decreased odds of improvement in community adjustment by 0.10.

**Table 3:** Results for community adjustment: VA GLA Healthcare System, 10/1/14–9/30/15.

Independent variables (*n* = 418)^+^	OR	CI	*p*
Tenant-based voucher	1.19	(0.56, 2.51)	0.65
Age	1.01	(0.99, 1.04)	0.19
Male	1.89	(0.77, 4.69)	0.17
Ethnicity			
Latino	1.06	(0.55, 2.06)	0.87
Race			
White	1.30	(0.79, 2.13)	0.30
Married or partnered	1.37	(0.68, 2.78)	0.38
Have children	0.67	(0.39, 1.14)	0.14
Education	0.99	(0.88, 1.11)	0.83
Employment			
Full-time employment	1.88	(0.94, 3.77)	0.08
Part-time employment	1.01	(0.54, 1.88)	0.99
Military	0.67	(0.15, 3.08)	0.61
Student	2.19	(0.65, 7.31)	0.20
Criminal history (yes)	1.25	(0.78, 2.02)	0.36
Homeless episodes	0.92	(0.81, 1.04)	0.20
Self-reported physical health	1.22	(0.97, 1.52)	0.09
Diagnostic category			
At least one mental health diagnosis	1.64	(0.81, 3.33)	0.17
At least one medical diagnosis	1.30	(0.63, 2.67)	0.48
At least one SUD	2.18	(0.78, 6.05)	0.14
Combination of medical and mental health diagnoses	1.56	(0.76, 3.18)	0.23
Combination of medical and SUD diagnoses*	3.55	(1.01, 12.45)	0.048
Combination of mental and SUD diagnoses	1.55	(0.62, 3.88)	0.34
Combination of medical, mental, and SUD diagnoses*	2.40	(0.98, 5.85)	0.06
Service utilization			
Primary care visits	1.01	(0.98, 1.04)	0.47
Emergency department admission	1.27	(0.75, 2.17)	0.38
Mental health visits	0.99	(0.98, <1.00)	0.17
HUD-VASH visits	1.00	(0.99, 1.01)	0.61
Outpatient SUD visits	1.00	(0.97, 1.03)	0.80
Mental health inpatient admission*	0.10	(0.02, 0.47)	0.00
Medical/surgical inpatient admission	0.87	(0.40, 1.93)	0.74

^+^Subjects with missing independent variables were dropped from analysis.

*Significant p < 0.05.

### Housing Stability

The housing stability model as a whole was not statistically significant (likelihood ratio χ^2^ = 39.52, *p* = 0.09, pseudo-*R* ^2^ = 0.20). However, two of the variables were significant: full-time employment at baseline and mental health visits (see **Table 4**). Being employed full-time at baseline was associated with increased odds of being stably housed (versus the combined middle and low categories) by a factor of 13.44, and mental health visits were associated with decreased odds of being stably housed by 0.97.

**Table 4:** Results for housing stability: VA GLA Healthcare System, 10/1/14–9/30/15.

Independent variables (*n* = 426)^+^	OR	CI	*p*
Tenant-based voucher	1.40	(0.28, 7.07)	0.68
Age	1.02	(0.97, 1.07)	0.40
Male	0.57	(0.10, 3.24)	0.53
Ethnicity			
Latino	1.37	(0.32, 5.87)	0.67
Race			
White	1.00	(0.34, 2.98)	1.00
Married or partnered	1.63	(0.29, 9.10)	0.58
Have children	0.57	(0.17, 1.90)	0.36
Education	1.05	(0.81, 1.35)	0.71
Employment			
Full-time employment*	13.44	(2.36, 76.65)	0.00
Part-time employment	3.53	(0.61, 20.28)	0.16
Military	3.56	(0.04, 302.50)	0.58
Student	3.65	(0.17, 77.15)	0.41
Criminal history (yes)	1.08	(0.35, 3.35)	0.90
Homeless episodes	0.82	(0.60, 1.10)	0.19
Self-reported physical health	0.74	(0.45, 1.23)	0.24
Diagnostic category			
At least one mental health diagnosis	2.09	(0.38, 11.55)	0.40
At least one medical diagnosis	1.86	(0.31, 11.10)	0.50
At least one SUD	4.36	(0.41, 46.68)	0.22
Combination of medical and mental health diagnoses	0.64	(0.12, 3.27)	0.59
Combination of medical and SUD diagnoses	0.22	(0.03, 1.66)	0.14
Combination of mental and SUD diagnoses	2.15	(0.20, 23.12)	0.53
Combination of medical, mental, and SUD diagnoses	1.61	(0.20, 12.72)	0.65
Service utilization			
Primary care visits	1.00	(0.95, 1.05)	0.87
Emergency department admission	3.27	(0.96, 11.11)	0.06
Mental health visits*	0.97	(0.95, <1.00)	0.02
HUD-VASH visits	1.00	(0.98, 1.02)	0.99
Outpatient SUD visits	1.05	(0.98, 1.13)	0.19
Mental health inpatient admission	0.28	(0.02, 3.42)	0.32
Medical/surgical inpatient admission	0.33	(0.07, 1.66)	0.18

^+^Subjects with missing independent variables were dropped from analysis.

*Significant p < 0.05.

### Employment

The employment model as a whole was statistically significant compared to the null model with no independent variables (likelihood ratio χ^2^ = 61.95, *p* < 0.017, pseudo-*R* ^2^ = 0.36; see **Table 5**). Our model examined employment on quarter 3 and, among other factors, controlled for employment at baseline (i.e., part-time and full-time employment). The model identified four significant variables: full-time employment at baseline, part-time employment at baseline, SUD visits, and mental health visits. Being employed full-time at baseline was associated with increased odds by a notable factor of 42.10, being employed part-time at baseline was associated with increased odds by a factor of 5.44, and SUD visits were associated with increased odds of good employment by a factor of 1.07. Mental health visits were associated with decreased odds of good employment outcomes by a factor of 0.97.

**Table 5:** Results for employment: VA GLA Healthcare System, 10/1/14–9/30/15.

Independent variables (*n* = 144)^+^	OR	CI	*p*
Cons	0.00	(0.00, 0.71)	0.04
Age	0.97	(0.92, 1.02)	0.19
Tenant-based voucher	12.04	(0.81, 179.02)	0.07
Ethnicity			
Latino	0.15	(0.02, 1.47)	0.10
Race			
White	2.72	(0.84, 8.78)	0.09
Married or partnered	1.22	(0.30, 4.88)	0.78
Have children	0.68	(0.19, 2.47)	0.56
Education	1.28	(0.96, 1.72)	0.10
Employment			
Full-time employment*	42.10	(7.75, 228.68)	0.00
Part-time employment*	5.44	(1.35, 21.98)	0.02
Military	16.41	(0.76, 352.22)	0.07
Student	0.88	(0.06, 13.91)	0.93
Criminal history (yes)	3.08	(0.92, 10.26)	0.07
Homeless episodes	1.01	(0.74, 1.37)	0.97
Self-reported physical health	1.46	(0.85, 2.51)	0.17
Service utilization			
Primary care visits	0.96	(0.88, 1.06)	0.44
Emergency department admission	0.50	(0.14, 1.75)	0.28
Mental health visits*	0.97	(0.94, <1.00)	0.048
HUD-VASH visits	0.98	(0.95, 1.01)	0.17
Outpatient SUD visits*	1.07	(1.01, 1.13)	0.03
Medical/surgical inpatient admission	0.99	(0.14, 7.10)	0.99

^+^Subjects with missing independent variables were dropped from analysis.

*Significant p < 0.05.

## Discussion

This study identified factors associated with change in community integration outcomes among formerly homeless veteran participants engaged in supportive housing. Similar to previous studies, we found that medical, psychiatric, and SUD diagnoses impacted community integration (33, 34) in three specific areas (i.e., change in community adjustment, housing stability status, and change in employment). Employment at baseline was positively associated with housing stability and employment outcomes 1 year after housing. We also found that SUD visits over the year increased the odds of good employment, and baseline combined medical and SUD diagnoses were positively associated with community adjustment. Further, salient in our findings was the role of mental health service use, which was associated with all three of our outcomes of interest (i.e., community adjustment, housing stability, and employment).

Most research on community integration of formerly homeless individuals has focused on those that have severe mental illness. In contrast, we examined formerly homeless individuals regardless of mental health diagnoses and analyzed whether mental health illness or use of mental health services was associated with integration. Our research complements previous findings by examining community integration among a broader cross-section of formerly homeless individuals.

For community adjustment, baseline combined medical and SUD diagnoses were positively associated, whereas having a mental health inpatient admission over the follow-up period was negatively associated, with improvements. The positive association between combined physical and SUD at baseline and improvements in community adjustment over 1 year may be the result of the better access to health and substance use services that permanent housing provides compared to homelessness. The negative association between mental health admissions over 1 year and improvements in community adjustment was expected because psychiatric hospitalizations—and their associated symptomatology—are fundamentally disruptive to community adjustment.

Housing stability was significantly associated with full-time employment at baseline and mental health visits over 1 year. Full-time employment likely assisted individuals in meeting rental obligations. We found that as the number of mental health visits increased over the year, housing stability decreased, likely because the number of visits reflected the severity of mental health symptoms.

Full-time employment at baseline, part-time employment at baseline, and SUD visits over 1 year were associated with increased OR of good employment outcomes. Individuals with employment at baseline likely continued to engage in employment 1 year after housing. The beneficial effects of SUD visits on employment may reflect the value of the VA’s programs to provide stability for individuals coping with these disorders. In contrast, mental health visits over the year were associated with poor employment outcomes, suggesting that those with mental illness struggle to find and/or maintain employment.

In these analyses, we considered 29 variables, but relatively few were associated with our outcomes of interest. The low amount of explained variation in our three models suggests that key determinants of community adjustment might be missed in secondary analyses of the VA’s databases. For example, the database does not capture neighborhood factors, including socioeconomic characteristics and proximity to community resources, which may function as moderators of community integration outcomes (20, 35, 36). In addition, the VA does not collect cognitive and motivational variables that are important for community integration (37).

This study had several limitations. First, the diagnoses recorded in the HOMES reflect data from case managers, most of whom have a mental health background (in social work) and less familiar with medical diagnoses; some medical diagnoses may be under-reported. Future studies could incorporate additional procedures to identify diagnoses, including the evaluation of laboratory data and validated scales for the assessment of psychiatric disorders. Second, our data are limited to 1 year of reporting after housing placement and only captured relatively early changes in community integration. It is possible that changes in community integration become more pronounced over the course of several years. The length of time housed may be particularly important for formerly homeless individuals who may be experiencing housing stability for the first time after lengthy periods of homelessness and who may be struggling with various challenges, including mental and physical health and/or substance use addiction. Future studies should look to incorporate a long-term approach by exploring potential differences in community integration by the length of residence in PSH. Third, our sample represents a small segment of the HUD-VASH (e.g., 1 year of the HUD-VASH data at GLA). Participants served at the GLA facility largely resided in Los Angeles County (87%). Many factors, including geography and employment industries, make Los Angeles unique and may result in poor translation of these data to other settings. Fourth, our study is limited to the HOMES and does not include measures of civic engagement, social support, or self-reported assessments of community integration (10, 11), which would enable investigations to encompass the construct of psychological integration. Participants’ subjective experience is a missing component of community integration and should be considered in future studies. Currently, the available databases that capture the VA homelessness do not include detailed assessments of self-reported community integration. However, efforts are currently under way to conduct detailed in-person interviews and standardized assessments [e.g., Ref. (38)] to obtain more comprehensive assessments of community integration. Fifth, missing data in the HOMES were a notable limitation and could have had significant implications for our outcomes of interest. We acknowledge that this limited our analyses and warrants future exploration of other measures of community integration, including non-paid work (e.g., volunteering) and VA service connection. Lastly, our study did not account for the potential influences of housing quality or neighborhood characteristics (39). Housing quality, including unit and building characteristics, has been identified as an important predictor of housing stability (40). Further, neighborhood quality may vary among the HUD-VASH recipients and can impact community integration (41). For instance, physical proximity to community resources, including transportation, stores, and recreational facilities, is positively correlated with an individual’s engagement with the local community (20, 35). Neighborhood safety and neighbor relations are also associated with integration (42). Future studies should include assessments of the impact of housing quality and neighborhood factors on the community integration of PSH participants.

Our study highlights the importance of mental health issues, as indicated by mental health service use, and suggests that utilization of mental health services is an important indicator of improvements in community adjustment, housing stability, and employment. As programs strive to address the housing needs of formerly homeless individuals, it is imperative to consider how to maximize an individual’s well-being and assure that they are leading satisfying and productive lives in their new places of residence. For one, vocational rehabilitation programs may be essential in facilitating employment opportunities for those struggling with mental illness. The VA’s vocational rehabilitation programs should be examined to assess if they are successfully engaging the HUD-VASH recipients. Second, mental health service utilization is associated with community integration outcomes. Consequently, in addition to medical diagnoses, case managers can use service utilization rates to identify participants that may warrant additional supports during their transition to independent living. Further, understanding how to improve community integration within the HUD-VASH will require the VA’s investment in data collection and analysis, which includes a broader range of variables to better understand post-housing experiences. Such data collection should include in-person data using both quantitative and qualitative research aimed at understanding participants’ abilities, motivations, and experiences as they relate to successful community integration.

## Author Contributions

MC conceived of the presented idea and carried out analysis with the support of corresponding authors. GH provided analytical support. SG and MG supervised the project. MC wrote the manuscript with input from AG, SG, and MG. All authors discussed the results and contributed to the final manuscript.

## Funding

Financial support was provided by Lynne Sagalyn and Gary Hack Department of Urban Studies and Planning Fund at Massachusetts Institute of Technology and the Greater Los Angeles Veteran Affairs Research Enhancement Award Program on Enhancing Community Integration for Homeless Veterans. Dr. Chinchilla was supported by the VA Office of Academic Affiliations through the Health Service Research and Development Post-Doctoral Fellowship. The contents do not represent the views of the U.S. Department of Veterans Affairs or the United States Government.

## Conflict of Interest Statement

The authors declare that the research was conducted in the absence of any commercial or financial relationships that could be construed as a potential conflict of interest.

The handling editor declared a past co-authorship with several of the authors GH and MG.
